# Supplementary material for: Pathogens spread by high-altitude windborne mosquitoes
Source: bioRxiv. 2024 Dec 26:2024.12.26.630351. Preprint. [Version 1] doi: 10.1101/2024.12.26.630351 (PMC11703268; doi:10.1101/2024.12.26.630351)
Supplement: 1 [file NIHPP2024.12.26.630351V1-supplement-1.pdf]

bioRxiv preprint doi: https://doi.org/10.1101/2024.12.26.630351; this version posted December 26, 2024. The copyright holder for this preprint (which was not certified by peer review) is the author/funder. This article is a US Government work. It is not subject to copyright under 17 USC 105 and is also made available for use under a CC0 license.

Supplemental Results & Discussion

In this study we included samples collected at five aerial stations across West Africa to address the question of infection of high-altitude windborne mosquitoes with pathogens. A total of 1,249 mosquitoes were collected on 432 standard panels but no mosquito was collected on 301 control panels, demonstrating that mosquitoes were collected at altitude. Larger number female mosquitoes was sampled in Ghana (656; Agogo and Wenchi) than in Mali (361; Bia, Kenieroba, and Thierola, Fig. S1). A comparison of the high-altitude mosquito and pathogen compositions among these sites is beyond the scope of the present study and will require a larger sample size from most sites. The results revealed that mosquitoes infected with mosquito-borne pathogens were found in all sites (not shown).

Mosquito composition in altitude (Fig. 1a, Table S1) is typical of biological samples in being dominated by few common species whilst most species are represented by moderately frequent species and by rare species (Anderwartha and Birch 1954). This distribution is probably a reflection of both the abundance of these species on the ground over large catchment area as well as their aptitude to engage in high altitude flight. Although most rare species are less abundant at altitude, some may represent incidental events. For example, the two *Ae. aegypti*, a diurnal species that are attracted to people, were collected on the same panel during the first month of operation of a newly trained team. Additional studies will ascertain the status of rare species.

Consistent with previous studies (Huestis et al. 2019, Yaro et al. 2022, Atieli et al. 2023), at altitude female mosquitoes predominated (85%) reflecting a general species- and genera- independent pattern, because no heterogeneity was detected (Table S2). This asymmetry likely reflects sexual fitness differential associated with long-range migration. Accordingly, upon landing females are likely to find suitable larval sites with equal or higher prospects for larval development success, whereas males need to locate virgin females where they might be scarce and compete with local males that are expected to be in similar density to females—same ratio as in their provenance site. Although, mating in a new locality is expected to also increase male fitness, but the expected fitness increase is smaller due to the additional costs of locating virgin females and successfully competing with local males. Because females are probably inseminated by males from their provenance site, this asymmetry in long-range migration should not have consequences on sex-specific geneflow (Barth et al. 2013).

Heterogeneity in the fraction of gravid and unfed females has been detected among species (Table S3). Because all gravid females had at least one exposure to vertebrate blood whereas some of the unfed females may have not had such exposure, it is of interest *Cx. perexiguus* exhibits the highest fraction of gravid females (65%), which could further contribute for its high infection rate with diverse pathogens. (main text).

Younger mosquitoes have longer remaining life span, but older mosquitoes are more likely to be infected. The ratio of infectiousness/exposed (here, "infected" is inclusive for all body parts) mosquitoes can be used to estimate the fraction of mosquitoes above the minimum age to develop a disseminated infection (Gillies 1954, Gillies and Wilkes 1965). In tropical areas plasmodia typically require ≥7 days post infection to mature their oocysts in the midgut wall (abdomen) and release sporozoites into the haemocoel that accumulate in the salivary glands (thorax), thus initiating a disseminated infection. The first blood meal typically takes place on the adult's second day (Gillies 1954, Gillies and Wilkes 1963, 1965), hence this ratio across all plasmodial species with at least 1 disseminated infection was 0.5 (0.031/0.062; Table 1), implying that half the population is ≥9 days old. This is 2.5 times the typical fraction of older mosquitoes that had taken ≥3 bloodmeals on vertebrate hosts (Gillies and Wilkes 1965), thus explaining, at least in part, the high infection rates in this collection. Whether pathogens increase aptitude of infected

bioRxiv preprint doi: https://doi.org/10.1101/2024.12.26.630351; this version posted December 26, 2024. The copyright holder for this preprint (which was not certified by peer review) is the author/funder. This article is a US Government work. It is not subject to copyright under 17 USC 105 and is also made available for use under a CC0 license.

mosquitoes to engage in high-altitude windborne migration remains to be answered (Lion et al. 2006, Martini et al. 2015, Poulin and Dutra 2021). Novel methods to track mosquitoes over large distances and methods to determine their provenance would help address key questions on these journeys.

Finally, we touch on the question, how was the spread of pathogens by mosquitoes at altitude not already widely recognized by epidemiologists? The high mobility and near-continuous distribution of human and domestic animals led to the assumption that they are the primary long-range movers of their pathogens including mosquito-borne pathogens, alternatives were disregarded without evaluation. Inferring windborne-mosquito spread was deemed possible only when outbreaks occurred outside the disease typical range, vertebrate movement was soundly ruled out, and wind direction and speed aligned with the nearest known source (Garrett-Jones 1950, 1956, 1962, Sellers et al. 1977, 1982, Pedgley 1983, Sellers and Maarouf 1990); conditions that greatly limit discovery of pathogen spread by windborne mosquitoes even if it was the most common mode of spread. Additionally, this epidemiological analysis was applied primarily to zoonotic pathogens affecting people and domestic animals, thus missing most sylvatic pathogens that circulate among wild animals and represent the largest share of mosquito-borne pathogens in Africa (Lehmann et al. 2023). Infection rates for flaviviruses, Plasmodia, and filariae were higher in abdominal tissues than in head/thorax dissections (Fig. 1b, Tables 1 and S1).

Figure S1. Sources of mosquitoes used in the present study among the aerial sampling stations by sex.

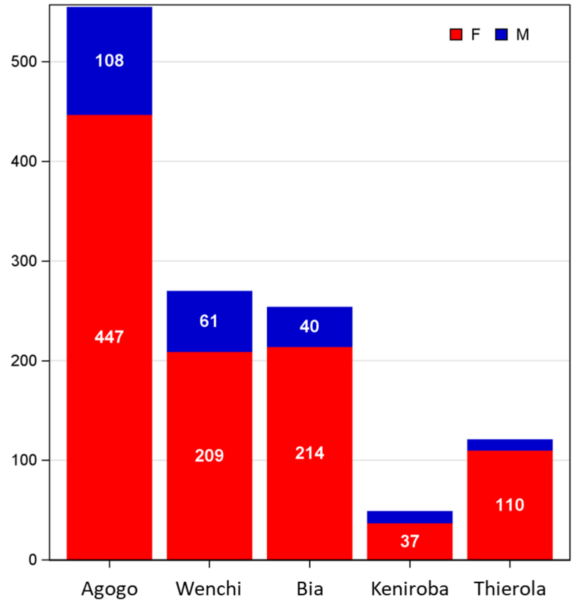

Figure S2. Bayesian phylogeny based on the mitochondrial *cytb* gene of plasmodia isolated from high-altitude mosquitoes (blue) compared with best-match sequences from MalAvi and NCBI databases (black). Posterior probabilities >0.85 are shown using a yellow circle. We grouped isolates separated by ≤3% sequence dissimilarity from closest neighbor to putative species and provisionally named them after the closest species or lineage in MalAvi that met this criterion (green boxes and fonts). Putative species that had no best match at this level were named sequentially, e.g., *P. mali sp. 1* after the country they were collected from. Note: *Haemoproteus* is closely related to *Plasmodium* but is vectored by non-mosquitoes biting flies.

bioRxiv preprint doi: https://doi.org/10.1101/2024.12.26.630351; this version posted December 26, 2024. The copyright holder for this preprint (which was not certified by peer review) is the author/funder. This article is a US Government work. It is not subject to copyright under 17 USC 105 and is also made available for use under a CC0 license.

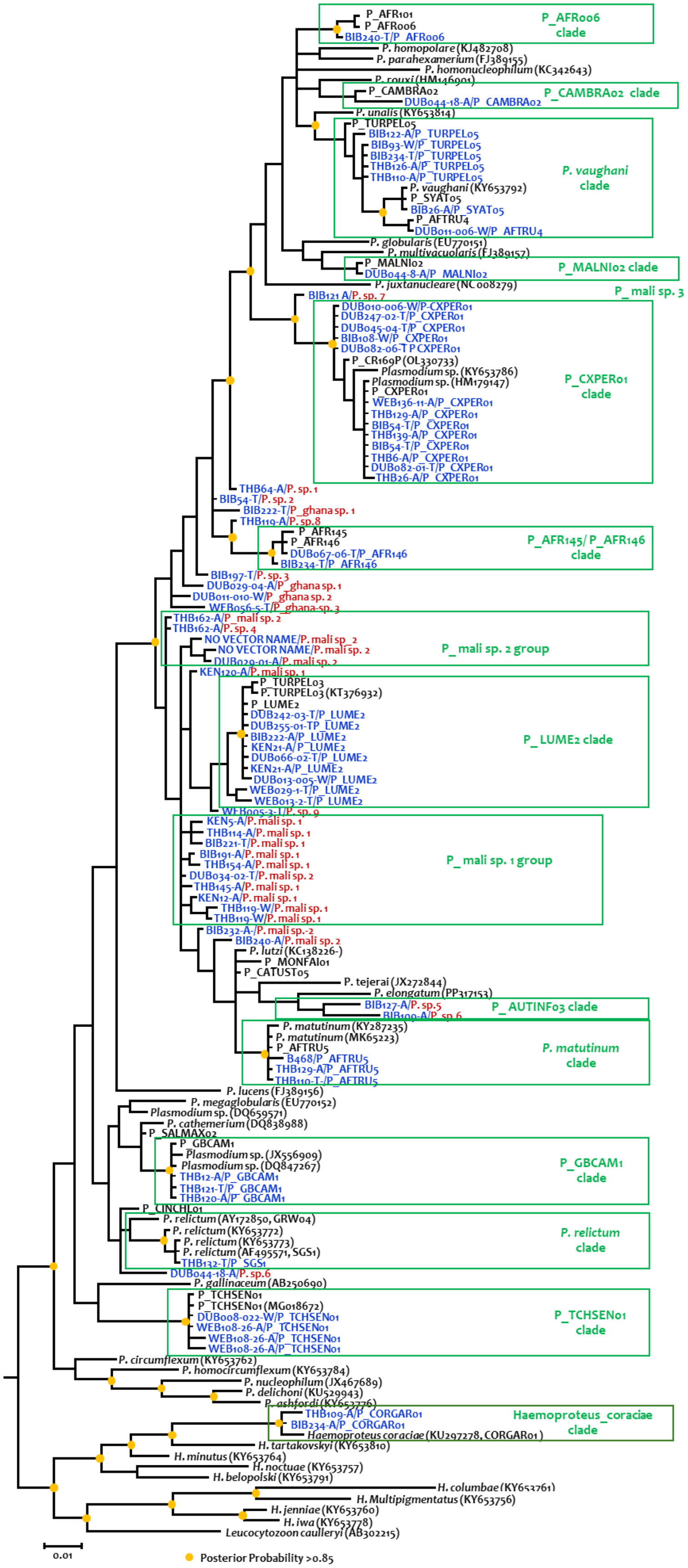

bioRxiv preprint doi: https://doi.org/10.1101/2024.12.26.630351; this version posted December 26, 2024. The copyright holder for this preprint (which was not certified by peer review) is the author/funder. This article is a US Government work. It is not subject to copyright under 17 USC 105 and is also made available for use under a CC0 license.

Figure S3. Overall infection rates per mosquito species (N≥5) based on pan-genus infection assays for flaviviruses (a), plasmodia (b), and filariae (c) with 90% CI. Higher infection rate (P<0.05, 2 side Binomial test) than the rate across all mosquito species (red lines) are indicated by stars.

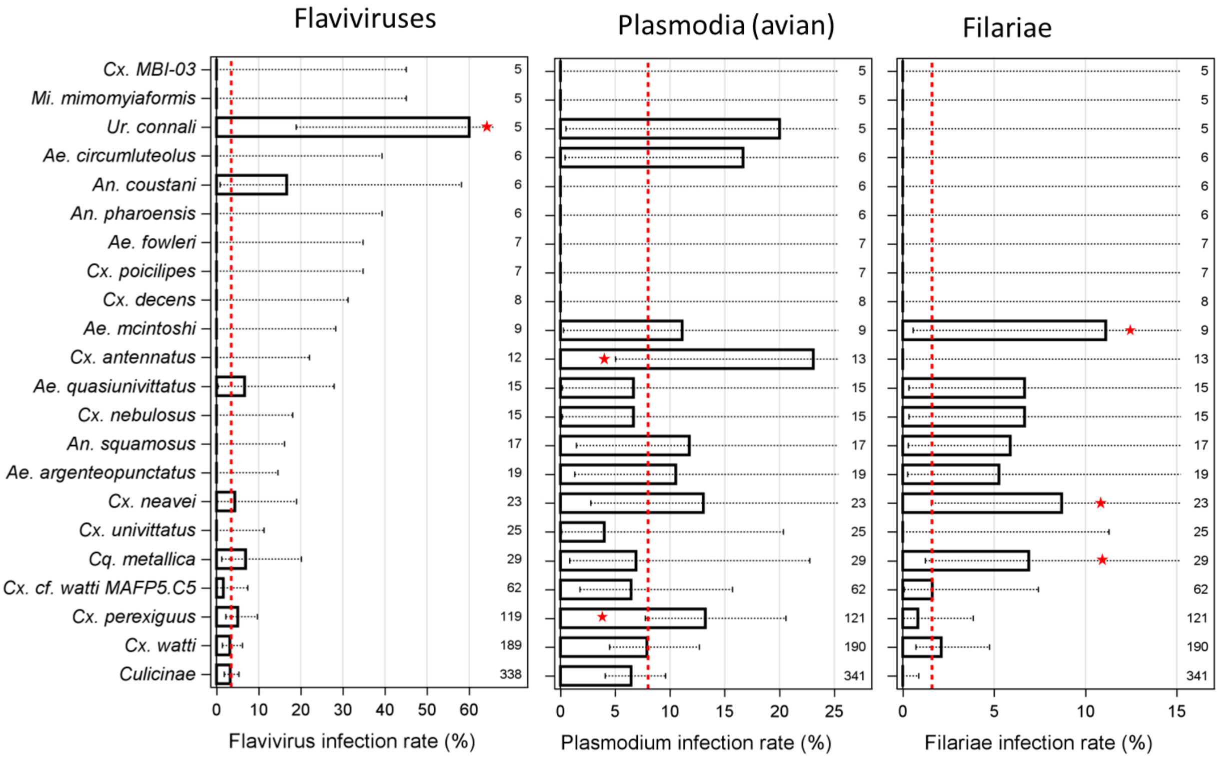

Figure S4. Relationship between disseminated (head-thorax) infection and exposure (abdominal) infection with plasmodia (a) and filariae (b) by species (abbreviated species name is listed with their sample size for infected species). Weighted linear regression (weighted by total sample size, blue) and 95% CI (gray) computed for species with N≥3 to identify outliers (red arrows) indicating competent vectors that typically feed preferentially on natural hosts, showing increased exposure.

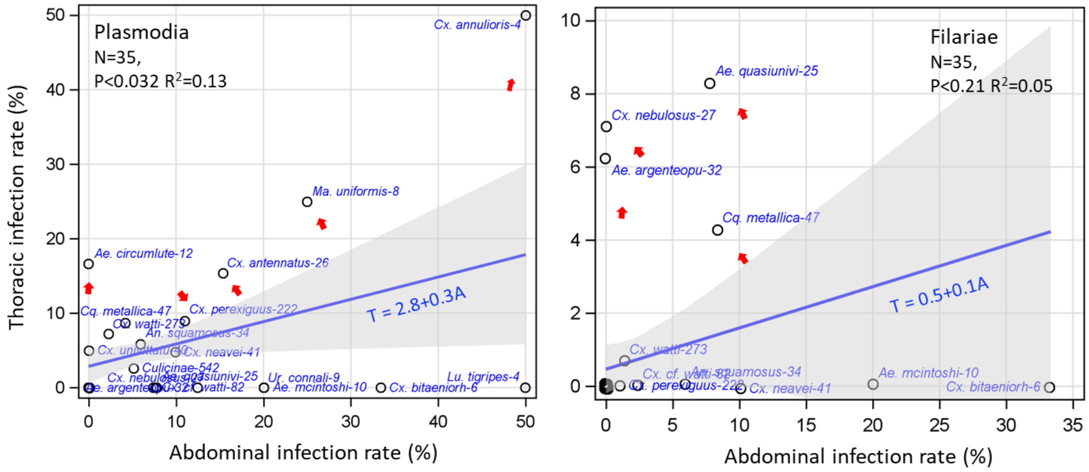

bioRxiv preprint doi: https://doi.org/10.1101/2024.12.26.630351; this version posted December 26, 2024. The copyright holder for this preprint (which was not certified by peer review) is the author/funder. This article is a US Government work. It is not subject to copyright under 17 USC 105 and is also made available for use under a CC0 license.

bioRxiv preprint doi: https://doi.org/10.1101/2024.12.26.630351; this version posted December 26, 2024. The copyright holder for this preprint (which was not certified by peer review) is the author/funder. This article is a US Government work. It is not subject to copyright under 17 USC 105 and is also made available for use under a CC0 license.

Figure S5. Effect of the species sample size on the number of pathogen species it is infected and infectious with. Loess regression lines with 95% CI describe the change in the number of pathogens species per mosquito species according to its ample size (log scale). Disseminated (head-thorax) infection (red) and total infection (blue) are shown. Abbreviated species name is listed for species with one or more pathogen species (black).

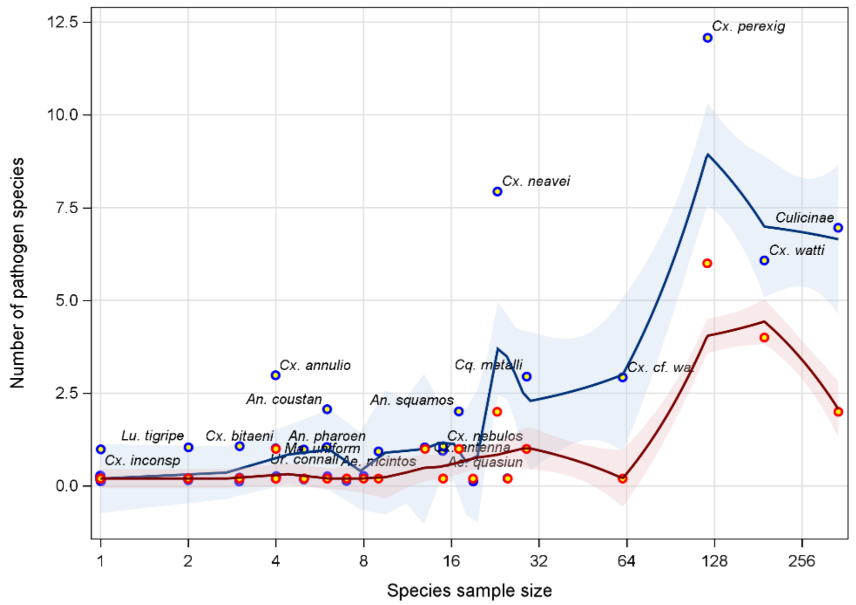

bioRxiv preprint doi: https://doi.org/10.1101/2024.12.26.630351; this version posted December 26, 2024. The copyright holder for this preprint (which was not certified by peer review) is the author/funder. This article is a US Government work. It is not subject to copyright under 17 USC 105 and is also made available for use under a CC0 license.

Supplementary Table 1. Composition of mosquitoes collected at altitude (120-290 m above ground; see main text)

Species	COUNT	Comments
Ae. aegypti	2	May represent contamination during panel processing: both specimens were collected on the same panel at the 1st month of operation of a new team
Ae. argenteopunctatus	19	
Ae. bromeliae	2	
Ae. circumluteolus	8	
Ae. dentatus	1	
Ae. fowleri	7	
Ae. mcintoshi	10	
Ae. quasiunivittatus	18	
Ae. sp. MBI-60	1	
Ae. sp. MBI-61	1	
Ae. sp. MBI-62	1	
An. cf. rivulorum	1	
An. coluzzii	2	
An. coustani	7	
An. funestus	1	May represent contamination during panel processing: a singe specimen that is recorded for the first time in altitude. Needs verification.
An. gambiae s.l.	3	May represent Anopheles coluzzii or Anopheles gambiae s.s. rather than a new species: molecular ID has failed repeatedly
An. gambiae s.s.	1	
An. pharoensis	6	
An. pretoriensis	1	
An. rufipes	3	
An. squamosus	20	
Anophelinae	2	May contain additional species
Cq. metallica	40	
Cq. sp. MBI-01	5	
Cq. sp. MBI-03	1	
Cx. MBI-03	6	
Cx. MBI-18	3	
Cx. annulioris	5	
Cx. antennatus	15	
Cx. bitaeniorhynchus	4	
Cx. cf. watti MAFP5.C5	80	
Cx. cinereus	1	
Cx. decens	9	
Cx. duttoni	1	
Cx. ghana sp. 1	3	
Cx. ghana sp. 2	3	
Cx. inconspicuosus	1	May be a different species but closely related. To be checked again
Cx. mali sp. 4	5	
Cx. neavei	28	
Cx. nebulosus	15	
Cx. perexiguus	147	
Cx. poicilipes	7	
Cx. sp. MBI-60	1	
Cx. sp. MBI-62	1	
Cx. sp. MBI-65	1	
Cx. sp. MBI-66	1	
Cx. sp. MBI-68	1	
Cx. sp. MBI-69	1	
Cx. univittatus	32	
Cx. univittatus B	4	
Cx. watti	212	
Er. sp. MBI-01	3	
Lu. tigripes	3	
Ma. africana	1	
Ma. sp. MBI-60	1	
Ma. uniformis	5	
Mi. mediolineata	2	
Mi. mimomyiaformis	5	
Ur. alboabdominalis	5	
Ur. connali	6	
Ur. ghana sp. 1	3	
Culicidae (unidentified)	5	May contain additional species
Culicinae (unidentified)	460	Failed to produce PCR prodcuts or quality mtCOI sequence. May contain additional
Total mosquitoes intercepted	1,249	Few preseved in 80% ethanol or on silica gel (not in RNAlater) were not subjected to RNA

bioRxiv preprint doi: https://doi.org/10.1101/2024.12.26.630351; this version posted December 26, 2024. The copyright holder for this preprint (which was not certified by peer review) is the author/funder. This article is a US Government work. It is not subject to copyright under 17 USC 105 and is also made available for use under a CC0 license.

Table S2: Sex composition by species in aerial collection (pooling species with N<10)

spMo2	Females % [N]	
Frequency<br>Percent<br>Row Pct	F	Total
Ae. argenteopunctatus	19<br>2.43<br>100.00	19<br>2.43
Ae. mcintoshi	9<br>1.15<br>90.00	10<br>1.28
Ae. quasiunivittatus	16<br>2.05<br>88.89	18<br>2.30
An. squamosus	17<br>2.17<br>85.00	20<br>2.56
Cq. metallica	30<br>3.84<br>75.00	40<br>5.12
Cx. antennatus	13<br>1.66<br>86.67	15<br>1.92
Cx. cf. watti MAFP5.C5	65<br>8.31<br>81.25	80<br>10.23
Cx. neavei	23<br>2.94<br>82.14	28<br>3.58
Cx. nebulosus	15<br>1.92<br>100.00	15<br>1.92
Cx. perexiguus	122<br>15.60<br>82.99	147<br>18.80
Cx. univittatus	25<br>3.20<br>78.13	32<br>4.09
Cx. watti	190<br>24.30<br>89.62	212<br>27.11
PooSpecies	122<br>15.60<br>83.56	146<br>18.67
Total	666<br>85.17	782<br>100.00
Statistic	DF	Value; Prob
Chi-Square	12	16.2; 0.1817
Likelihood Ratio Chi-Square	12	20.9; 0.0522
Monte Carlo Estimate for the Exact Test		
Pr >= ChiSq	**0.0759**	
99% Lower Conf Limit	0.0691	
99% Upper Conf Limit	0.0827	
Sample Size	782	
Number of Samples	10000	

bioRxiv preprint doi: https://doi.org/10.1101/2024.12.26.630351; this version posted December 26, 2024. The copyright holder for this preprint (which was not certified by peer review) is the author/funder. This article is a US Government work. It is not subject to copyright under 17 USC 105 and is also made available for use under a CC0 license.

Table S3: Fraction of gravid females in aerial collection by species (species with N<10 pooled)

Species	Gravid fraction % [N]		
Frequency<br>Percent<br>Row Pct	Gravid %	Total	
Ae. argenteopunctatus	8<br>1.23<br>42.11	19<br>2.93	
Ae. mcintoshi	3<br>0.46<br>42.86	7<br>1.08	
Ae. quasiunivittatus	9<br>1.39<br>52.94	17<br>2.62	
An. squamosus	11<br>1.70<br>64.71	17<br>2.62	
Cq. metallica	12<br>1.85<br>41.38	29<br>4.48	
Cx. antennatus	7<br>1.08<br>53.85	13<br>2.01	
Cx. cf. watti MAFP5.C5	20<br>3.09<br>33.90	59<br>9.10	
Cx. neavei	15<br>2.31<br>65.22	23<br>3.55	
Cx. nebulosus	6<br>0.93<br>42.86	14<br>2.16	
Cx. perexiguus	80<br>12.35<br>65.04	123<br>18.98	
Cx. univittatus	7<br>1.08<br>28.00	25<br>3.86	
Cx. watti	60<br>9.26<br>32.26	186<br>28.70	
PooSpecies	47<br>7.25<br>40.52	116<br>17.90	
Total	285<br>43.98	648<br>100.00	
Chi-Square Test			
Statistic	DF	Value	Prob
Chi-Square	12	46.4659	<.0001
Likelihood Ratio Chi-Squa	12	46.8317	<.0001
Monte Carlo Estimate for the Exact Test			
Pr >= ChiSq	0.0001		
99% Lower Conf Limit	<.0001		
99% Upper Conf Limit	0.0004		
Number of Samples	10000		
Sample Size = 648	648		

bioRxiv preprint doi: https://doi.org/10.1101/2024.12.26.630351; this version posted December 26, 2024. The copyright holder for this preprint (which was not certified by peer review) is the author/funder. This article is a US Government work. It is not subject to copyright under 17 USC 105 and is also made available for use under a CC0 license.

Table S4. Infection rates of mosquitoes intercepted at altitude (120-290 m above ground) with insect-specific viruses and with non-mosquito-borne pathogens.

Pathogen	Overall[a] (N)	Method	Nat. Host/Vector
**Insect specific flaviviruses**	1.1% (11/1,002)	Pan-Flavivirus PCR	Mosquito-specific
Barkedji virus	0.1% (1/1,002)	Metagenomics	Mosquito-specific
Anopheles flavivirus variant 1	0.1% (1/1,002)	Metagenomics	Mosquito-specific
Flavivirus-like endogenous virus	0.2% (2/1,002)	Metagenomics + Sanger	Mosquito-specific
Nienokue virus	0.5% (5/1,002)	Metagenomics + Sanger	Mosquito-specific
Mercado virus	0.1% (1/1,002)	Metagenomics	Mosquito-specific
Quang Binh virus	0.1%(1/1,002)	Metagenomics	Mosquito-specific
**Other Insect-specific viruses[d]**			
Hameenlinna phasivirus	2.1% (1/48)	Metagenomics	Mosquito-specific
Aedes aegypti To virus 2	4.2% (2/48)	Metagenomics	Mosquito-specific
Zeya Brooke Chaq-like virus	2.1% (1/48)	Metagenomics	Mosquito-specific
Verdadero virus	2.1% (1/48)	Metagenomics	Mosquito-specific
Spilikins virus	2.1% (1/48)	Metagenomics	Mosquito-specific
Orbis virgavirus	2.1% (1/48)	Metagenomics	Mosquito-specific
Mos8Chu0 chuvirus	2.1% (1/48)	Metagenomics	Mosquito-specific
Culex mosquito virus 4	2.1% (1/48)	Metagenomics	Mosquito-specific
Broome reo-like virus 1	2.1% (1/48)	Metagenomics	Mosquito-specific
XiangYun mono-chu-like virus 7	6.3% (3/48)	Metagenomics	Mosquito-specific
Aedes binegev-like virus 2	2.1% (1/48)	Metagenomics	Mosquito-specific
Osterfarnebo virus	4.2% (2/48)	Metagenomics	Mosquito-specific
Culex pipiens-associated Tunisia virus	2.1% (1/48)	Metagenomics	Mosquito-specific
Mole Culex virus	2.1% (1/48)	Metagenomics	Mosquito-specific
Gysinge virus	4.2% (2/48)	Metagenomics	Mosquito-specific
Environmental Rhabdovirus	2.1% (1/48)	Metagenomics	Mosquito-specific
Biggie virus	2.1% (1/48)	Metagenomics	Mosquito-specific
Atrato-partiti like virus 1	2.1% (1/48)	Metagenomics	Mosquito-specific
Hubei virga-like virus 2	2.1% (1/48)	Metagenomics	Mosquito-specific
Forneby virus	2.1% (1/48)	Metagenomics	Mosquito-specific
Zhejiang mosquito virus 3	2.1% (1/48)	Metagenomics	Mosquito-specific
**Haemosproida (excluding Plasmodium spp.)**			Vertebrates/Biting midges
- Haemoproteus (parahaemoproteus) coraciae	0.2% (2/1006)	Cox sequence	
**Trypanosomatida**			
- Trypanosoma theileri (100% & 97.6% similarity)	6.3 (3/48)	Metagenomics	Bovids/Cervids/biting flies
- Paratrypanosoma confusum (100% similarity)	14.6 (7/48)	Metagenomics	Bovids/Cervids/biting flies
- Trypanosoma sp. 1	2.1% (1/48)	Metagenomics	Vertebrates/arthropods
- Trypanosoma sp. 2	2.1% (1/48)	Metagenomics	Vertebrates/arthropods

bioRxiv preprint doi: https://doi.org/10.1101/2024.12.26.630351; this version posted December 26, 2024. The copyright holder for this preprint (which was not certified by peer review) is the author/funder. This article is a US Government work. It is not subject to copyright under 17 USC 105 and is also made available for use under a CC0 license.

Table S5. Primers used for pathogen and mosquito detection and identification.

Target organism	Gene	Primers/Probe	Sequence 5'-3'	Type of PCR	Sequenced amplicon size (bp)	Tm (°C)	Ref
		Flavi_F	TACAACATGATGGGAAAGAGAGAGAARAA				Vina-Rodriguez et al 2017
Flaviviridae		Flavi_R	GTGTCCCAKCCRGCTGTGTCATC	RT-qPCR with melting curve	NA	NA	
		Alpha_F	GIAAYTGYAAYGTIACICARATG				Vina-Rodriguez et al 2017
Alphaviridae		Alpha_R	GCRAAIARIGCIGCIGCYTYIGGICC		NA	NA	
		FLAVI1-F	GCATCTAYAWCAYNATGGG				
		FLAVI1-R	CCANACNYNRTTCCANAC	Nested PCR			Vazquez et al., 2012)
		FLAVI2-F	GCNATNTGGTWYATGTGG				
Flaviviridae		FLAVI2-R	CATRTCTTCNGTNGTCATCC		**~960 bp**	50°C – 3 min	
		Plasmo_cox_15_F	AGGAACTCGACTGGCCTACA				
		Plasmo_cox_16_R	CCAGCGACAGCGGTTATACT	qPCR			Mediannikov et al. 2013)
Plasmodium		Plasmo-cox_P	6FAM- CGAACGCTTTTAACGCCTGACATGG -TAMRA			NA	
		HAEMF	ATGGTGCTTTCGATATATGCATG				
		HAEMR2	GCATTATCTGGATGTGATAATGGT				Hellgreen et al. 2004;
		HaemNFI	CATATATTAAGAGAAITATGGAG				
Plasmodium		HaemNR3	ATAGAAAGATAAGAAATACCATTC	Nested PCR	477		
	mt-Cyt B	DW4	TGTTTGCTTGGGAGCTGTAATCATAATGTG				
	mt-Cyt B	DW2	TAATGCCTAGACGTATTCCTGATTATCCAG				Templeton et al 2016
	mt-Cyt B	NCYBINR	CTTGTGGTAATTGACATCCAATCC				
Plasmodium	mt-Cyt B	NCYBINF	TAAGAGAATTATGGAGTGGATGGTG		799		
		qFil-28S-F	TTGTTTGAGATTGCAGCCCA				
		qFil-28S-R	GTTTCCATCTCAGCGGTTTC	qPCR		NA	
Filariae		qFil-28S-P	6FAM-CAAGTACCGTGAGGGAAAGT-TAMRA				Laidoudi et al. 2020
		Fwd.957	ATRGTTTATCAGTCTTTTTTTATTGG	PCR			
		Rwd.1465	GCAATYCAAATAGAAGCAAAAGT		509	52	
Insects	mt-COI	LCO	GGTCAACAAATCATAAAGATATTGG	PCR			Folmer et al 1994
		HCO	TAAACTTCAGGGTGACCAAAAAATCA		658	48C for 30sec	
